# Detection Method for Gene Doping in a Mouse Model Expressing Human Erythropoietin from Adeno-Associated Virus Vector-9

**DOI:** 10.3390/genes15060709

**Published:** 2024-05-29

**Authors:** Takehito Sugasawa, Atsushi Hirokawa, Norihiro Otani, Yasuharu Kanki, Kieu DM Nguyen, Tohru Takemasa, Koichi Watanabe, Yoshinori Takeuchi, Naoya Yahagi, Yoichiro Takahashi

**Affiliations:** 1 Laboratory of Clinical Examination and Sports Medicine, Department of Clinical Medicine, Institute of Medicine, University of Tsukuba, 1-1-1 Tennodai, Tsukuba 305-8577, Japan; 2 Department of Sports Medicine Analysis, Open Facility Network Office, Organization for Open Facility Initiatives, University of Tsukuba, 1-1-1 Tennodai, Tsukuba 305-8577, Japan; 3 College of Medicine, School of Medicine and Health Sciences, University of Tsukuba, 1-1-1 Tennodai, Tsukuba 305-8577, Japan; 4 Human Biology Program, University of Tsukuba, 1-1-1 Tennodai, Tsukuba 305-8577, Japan; 5 Institute of Health and Sport Sciences, University of Tsukuba, 1-1-1 Tennodai, Tsukuba 305-8574, Japan; takemasa.tohru.gm@u.tsukuba.ac.jp (T.T.);; 6 Division of Endocrinology and Metabolism, Department of Medicine, Jichi Medical University, Shimotsuke 329-0498, Japan; 7 Department of Legal Medicine, Institute of Medicine, University of Tsukuba, 1-1-1 Tennodai, Tsukuba 305-8575, Japan

**Keywords:** gene doping, erythropoietin, mouse model, RNA-seq, TaqMan-qPCR assay

## Abstract

With the rapid development of gene therapy technology in recent years, its abuse as a method of sports doping in athletics has become a concern. However, there is still room for improvement in gene-doping testing methods, and a robust animal model needs to be developed. Therefore, the purposes of this study were to establish a model of gene doping using recombinant adeno-associated virus vector-9, including the human erythropoietin gene (rAAV9-h*EPO*), and to establish a relevant testing method. First, it was attempted to establish the model using rAAV9-h*EPO* on mice. The results showed a significant increase in erythrocyte volume accompanied by an increase in spleen weight, confirming the validity of the model. Next, we attempted to detect proof of gene doping by targeting DNA and RNA. Direct proof of gene doping was detected using a TaqMan-qPCR assay with certain primers/probes. In addition, some indirect proof was identified in RNAs through the combination of a TB Green qPCR assay with RNA sequencing. Taken together, these results could provide the foundation for an effective test for gene doping in human athletes in the future.

## 1. Introduction

The act of doping, as defined by the World Anti-Doping Agency (WADA) and as evidenced by numerous instances of doping in professional and Olympic sports, involves the use of prohibited substances and/or methods in sports to enhance an athlete’s physical performance and overall success [1]. The objective of doping is to improve an athlete’s physical performance. In 1999, the WADA was established with the objective of promoting clean sports by conducting scientific research on doping, educating athletes and the public about anti-doping measures, developing anti-doping strategies, and monitoring the World Anti-Doping Code to ensure soundness and fairness in sports worldwide [2]. Nevertheless, despite the considerable efforts of WADA, doping has not been eradicated from competitive sports.

In fact, at the Tokyo 2020 Olympics in 2021, a track and field athlete was arrested for blood doping [3]. Advances in medicine and science are known to make doping more sophisticated. Therefore, we must continue to work toward establishing testing methods for any form of doping in order to protect the fairness and health of athletes.

In recent years, gene therapy technology has been developing rapidly. In particular, the vaccine against the coronavirus disease 2019 (COVID-19) virus that shook the world is an application of this gene therapy technology. In addition, around the world, gene therapies using various vectors have been approved for patients who have muscular dystrophy, spinal muscular atrophy (SMA), hemophilia, and various other diseases [4,5]. These diseases used to be incurable, but technological innovations in gene therapy have saved patients’ lives, and it is no exaggeration to say that this is the most advanced form of medicine this century.

Gene therapy technology is evolving at this very moment, but there remains a possibility that it may be abused in the form of gene doping, and therefore, there is an urgent need to develop a method of testing for its presence. In fact, WADA, which oversees the world’s anti-doping testing, has also been wary of gene doping.

The 2024 version of the International Standard Prohibited List [6], which forms part of the World Anti-Doping Code 2021 [7], is published and updated annually by WADA. This list includes a number of doping methods, one of which is “gene doping”. This form of doping involves the use of gene therapy technology in an illicit manner. Our research group has been conducting research for more than seven years to develop a detection method for gene doping [8,9,10,11,12].

Erythropoietin (EPO) has a long history of use in doping [13,14,15,16,17] and is the substance causing the most alarm. In addition, recombinant adeno-associated viral (rAAV) vectors have been confirmed to be safe in gene therapy [18] and are frequently used in gene therapy clinical trials [19]. Therefore, the combination of erythropoietin and rAAV is considered to be at high risk of abuse.

Furthermore, fluctuations in RNA expression in whole-blood samples have been proposed in our previous study as a means of indirect proof for inclusion in the Athlete Biological Passport (ABP) [8]. The fundamental principle underlying the ABP is to monitor a select number of biological variables, which indirectly reveal the effects of doping over time. This approach contrasts with the traditional method of attempting to detect the doping substance or method itself [20].

Therefore, regular observation of RNA expression changes in whole blood could be added as a parameter for the ABP in the future. In fact, our previous studies with a gene doping model have shown the advantage of quantifying the variability of RNA expression in whole blood [8].

Furthermore, WADA explains that the ABP can be used to identify athletes requiring further attention through intelligent, timely interpretation of passport data and can notably be used as a complement to analytical methods to further refine and strengthen overall anti-doping strategies [20]. Therefore, in this study, total RNA sequencing (RNA-seq) using whole-blood RNA was also performed to identify novel indirect proof based on the concept of the ABP.

In summary, the objectives of this study were to establish a model of gene doping using adeno-associated virus vector-9, including the human erythropoietin gene (rAAV9-h*EPO*), and to establish a method for detecting direct or indirect evidence of genetic doping using blood. As a result, we were able to successfully create the mouse model and prove that it is possible to accurately detect gene doping.

## 2. Materials and Methods

### 2.1. Creation of the rAAV9-hEPO Vector

To establish a robust gene-doping mouse model injected with rAAV9-h*EPO*, a viral vector was created via the following procedures using an outsourcing company (VectorBuilder Inc., Science City, Guangzhou, China). The pAAV expression vector containing 5′ ITRs (inverted terminal repeats) and 3′ ITRs was created with the VectorBuilder web tool. The plasmid was loaded with elements of CMVp (Human cytomegalovirus immediate early enhancer/promoter), h*EPO*, and WPRE (Woodchuck hepatitis virus posttranscriptional regulatory element). After that, we requested VectorBuilder to create rAAV9 using the plasmid and to obtain the virus particles by an amplification and purification method using the ultra-centrifugal method. The titers of the virus particles obtained were 2.76 × 10^13^ vg (viral genome)/mL.

### 2.2. Animal Experiments

All animal experiments conducted in this study were approved by the Animal Care Committee of the University of Tsukuba (approval number 22-125). The mice were obtained from CREA Japan (Meguro, Tokyo, Japan) at the age of six weeks and then underwent a one-week acclimation period. The mice were bred and maintained under specific pathogen-free conditions in an air-conditioned animal house. The animals were subjected to a 12/12 h light/dark cycle with standard mouse pellets and water provided ad libitum. Upon commencement of the experiments, the mice were 7 weeks old and weighed 35.6 g ± 1.7 (average ± SD). The experiments were conducted in two phases, namely, short-term and long-term.

**Figure 1:**
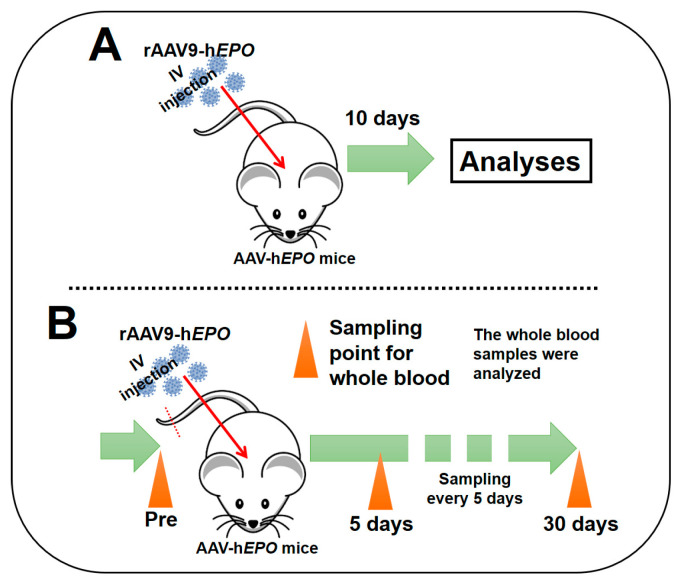
An overview of animal experiments. (**A**) short-term experiments; (**B**) long-term experiments, including repeated sampling.

The initial objective of the short-term experiments was to establish a model of gene doping in mice by administering a vectorized form of human *EPO* via a recombinant adeno-associated virus (AAV). An overview of the experimental approach is depicted in Figure 1A, which presents the timeline of the study and a description of the experimental design. After one week of acclimatization, the mice were randomly assigned to the control group (n = 8; designated as “Con”) or the rAAV9-h*EPO* group (n = 8; designated as “AAV-h*EPO*”). Mice in the AAV-h*EPO* group received an intraorbital sinus injection of the rAAV9-h*EPO* vector at a dose of 1011 vg/100 µL/mouse under the administration of systemic isoflurane anesthesia. The mice in the control group (n = 8) received injections of the 10% glycerol/PBS buffer (100 µL/mouse) used to suspend the rAAV9-h*EPO* vector. Ten days after the injection, whole blood was obtained from the inferior vena cava with EDTA-2Na as an anticoagulant under systemic isoflurane inhalation anesthesia. The mice were then euthanized. The collected whole-blood samples were subjected to a series of preprocessing procedures, including separation of plasma, aliquoting, and so forth, for the purpose of facilitating subsequent analyses. Samples of the liver and spleen were also harvested and rapidly frozen in liquid nitrogen until further analysis. Additionally, other samples of spleen and liver tissues underwent immersion into a 10% formalin neutral buffer solution overnight in order to yield paraffin block specimens.

Long-term experiments, including repeated sampling, were conducted to investigate the duration of direct and indirect proof of gene doping that could be positively detected from approximately one drop of whole blood. An overview of these experiments is shown in Figure 1B. After an initial acclimatization period of one week, small whole-blood samples (approximately 100 µL; two drops) were collected from 11 mice using EDTA-2Na with cuts approximately 1 mm distant from the tail tip (referred to as the “Pre” time point). The AAV9-h*EPO* vector was subsequently injected using the methods previously described. Subsequently, blood samples were collected at regular intervals over a 30-day period following the injection, as illustrated in Figure 1B. In order to minimize the adverse effects of continuous blood collection on mice, including anemia and hematopoietic stimulation, a method was employed that only obtained a small amount (approximately 100 µL) from the tail tip. The collected blood samples were divided into two samples of approximately 50 µL (equivalent to approximately one drop) each. DNA and RNA were extracted from these samples and subjected to further analysis.

The animal experimental methods described so far are identical in many respects to those of our previous study [8]. In addition, the description of this later experimental method is likewise similar in many respects to the experimental methods of our previous study [8]. Therefore, the method descriptions that follow may cite reference number 8.

### 2.3. Measurements of General Hematopoietic Markers

Hematological indicators of red blood cell (RBC) count, hemoglobin (HGB) level, and hematocrit (HCT) value obtained from whole blood in the short-term experiments were measured on an automatic blood analyzer (Celltac α MEK6458; NIHON KODEN, Shinjuku, Tokyo, Japan) using 50 µL of whole blood [8]. Whole-blood volumes were also measured using a 5 mL syringe during the whole-blood collection.

### 2.4. Enzyme-Linked Immunosorbent Assay

An enzyme-linked immunosorbent assay (ELISA) was performed to confirm hEPO secretion from the liver into the blood of short-term AAV-h*EPO* mice. Anti-hEPO (Cat# 500-P318; PeproTech, Cranbury, NJ, USA) was diluted to 0.5 µg/mL in PBS as a capture antibody, and 100 µL of the antibody solution was applied to ELISA plates for triplicate measurements. The plate was incubated overnight at 4 °C and then washed four times with PBS containing 0.05% Tween 20 (PBS-T). A total of 100 µL of plasma samples diluted 20-fold with PBS were added to the wells and incubated for 1 h at room temperature. Solutions of recombinant hEPO protein (Cat# 100-64; PeproTech) in PBS containing 5% BSA were also added to generate a hEPO standard curve between 100 ng/mL and 195 pg/mL. The wells were then incubated for 1 h at room temperature with a 100 µL solution of a detection antibody (biotinylated anti-hEPO; Cat# 500-P318BT; PeproTech) at a concentration of 0.25 ng/mL. After incubation, the plate was washed in the same manner, and a 100 µL solution of HRP-conjugated streptavidin (Cat# SA00001-0; Proteintech, Rosemont, IL, USA) diluted 5000-fold with PBS-T was added to the wells. The plate was incubated for 30 min at room temperature and washed in the same manner. After washing, a 100 μL mixture of color reagent and substrate (ELISA POD Substrate A.B.T.S Kit, Cat# 14351-80; Nacalai Tesque, Nakagyo, Kyoto, Japan) was applied and incubated for 15 min, followed by the application of a 100 μL stop reagent for color reaction. Finally, the absorbance at 405 nm was measured with a reference at 600 nm using a microplate reader. Using the absorbance data, a 4-parameter logistic regression standard curve of the Rodbard equation was generated in ImageJ Fiji (Life-Line version, v1.53q), and the plasma hEPO concentration was calculated in duplicates based on the standard curve (R^2^ = 0.99) [8].

### 2.5. TB Green qPCR Assay for Tissue

A TB Green qPCR assay was performed as an intercalation method to confirm the expression of hematopoietic marker genes in the liver and spleen in the short-term trial. RNAiso Plus (Cat# 9180; Takara Bio, Kusatsu, Shiga, Japan) was used to extract RNA from the liver and spleen according to the manufacturer’s instructions. The extracted RNA solution was diluted in Milli-Q water (Merck Millipore, Burlington, MA, USA) and adjusted to a concentration of 100 ng/µL. Then, 500 ng of RNA was used to prepare cDNA using PrimeScript RT Master Mix (Cat# RR036A; Takara Bio) according to the manufacturer’s instructions. The cDNAs were diluted 10× with Milli-Q water and subjected to quantitative real-time PCR (qPCR) based on intercalated fluorescent dye. For quantification of gene expression of hematological markers in the liver and spleen, qPCR was performed using TB Green Premix Ex Taq II (Cat. No. RR820; Takara Bio) with QuantStudio 5 PCR Systems (Thermo Fisher Scientific, Waltham, MA, USA) in duplicate measurements. The target gene list and primer sequences are shown in Supplementary Table S1. Template volumes and primer concentrations were 2 µL and 100 nM, respectively, for a total reaction volume of 10 µL per well. Negative control wells were also set up using Milli-Q water instead of a template. Thermal cycling conditions were 95 °C for 5 min, followed by 40 cycles of 95 °C for 2 s and 60 °C for 20 s, and a melting curve step. The ΔΔCt method was then used to calculate relative gene expression values with respect to 18S rRNA [8].

### 2.6. Histology

The paraffin blocks of each tissue obtained were sectioned into 3 μm thick slices, and H&E (hematoxylin and eosin) staining was performed. After the staining, the morphology was observed under a microscope.

### 2.7. DNA Extraction from Whole-Blood Samples

A Maxwell RSC Blood DNA Kit (Cat# AS1400; Promega, Madison, WI, USA) was used to extract total DNA from 100 µL (short-term experiment) or 50 µL (long-term experiment) of whole blood, using a Maxwell RSC Instrument (Promega), according to the manufacturer’s instructions. The DNAs were dissolved in 50 µL of Milli-Q Water, and the solutions were subjected to the TaqMan-qPCR assays.

### 2.8. TaqMan qPCR Assay

A TaqMan qPCR assay was performed on the whole-blood DNA. For the detection of direct proof of gene doping in whole-blood DNA in the short- and long-term experiments, the primers and TaqMan probes were designed to target the *EPO* gene (2 types), WPRE (Woodchuck hepatitis virus posttranscriptional regulatory element named Pr-WPRE), and CMVp (cytomegalovirus promoter named Pr-CMVp) to ensure specific amplification of the rAAV9-h*EPO* genome using the Primer-BLAST web tool [21]. The primers and TaqMan probes for the two types of h*EPO* genes were designed with exon–exon junctions (exons 2–4 and 4–5; primer names are Pr-hEPO-1 and Pr-hEPO-2), which are considered non-amplifying structures in the human genome. This strategy of designing primers/probes for the h*EPO* gene to detect gene doping follows WADA’s guidelines [22]. The primers and probes were also tested for specificity by in silico PCR using Pri-mer-BLAST; these evaluations confirmed the absence of amplification in the human and mouse genomes and the absence of amplification in the mouse genome. The sequences of the primers and TaqMan probes are shown in Supplementary Table S1. Primers and probes were synthesized in a double-quencher system by Integrated DNA Technologies (IDT; Coralville, IA, USA). The TaqMan qPCR assay was then performed in duplicate measurements to detect direct detection in whole blood DNA by absolute quantification. PrimeTime Gene Expression Master Mix (Cat# 1055771; IDT) was used with the primers and TaqMan probes on QuantStudio 5 Real-Time PCR Systems (Thermo Fisher Scientific, Waltham, MA, USA). The template DNA volume was 2 µL, and the primer and probe concentrations were 200 nM and 200 nM, respectively, for a total reaction volume of 10 µL per well. Milli-Q water was used instead of a template for negative control wells. DNA from 6 human cell lines (fibroblast, HK2 cell, Caco-2 cell, HEK293 cell, mesenchymal stem cell, and HepG2 cell) was used as a negative control. pAAV-expressing vectors containing the h*EPO* gene, WPRE, and CMVp (pAAV-h*EPO*) at 10 pg/μL were used to construct a standard curve for absolute quantification, and the range of the standard curves was set at 0.95 to 1.86 × 10^6^ copies/μL for the pAAV. The thermal cycling conditions for all primer/probe pairs were 95 °C for 5 min, followed by 40 cycles of 95 °C for 2 s and 60 °C for 20 s. All standard curves had R^2^ > 0.99 [8].

### 2.9. Sanger Sequencing

The amplicon-containing solutions were pooled in a 1.5 mL microtube and electrophoresed on a 2% agarose gel after TaqMan qPCR. The DNA amplicon bands were visualized using an ethidium bromide solution. The PCR amplicons were then purified using a NucleoSpin Gel and PCR Clean-up Kit (Cat# 740609; Takara Bio, Kusatsu, Shiga, Japan). To verify the sequence of the DNA amplicons, 5 ng of the purified DNA was then subjected to Sanger sequencing. Sanger sequencing was outsourced to an external company (GENEWIZ, Shinagawa, Tokyo, Japan). BioEdit ver. 7.2.5 was used to analyze the Sanger sequencing data. 7.2.5 (developer: Tom Hall, Carlsbad, CA, USA) [8]. Alignment analysis was performed against the DNA sequence of rAAV9-h*EPO* for 42 nucleotides obtained from the Sanger sequence.

### 2.10. Total RNA-Seq

Based on the concept of ABP [20], total RNA-seq was performed to identify RNA as a novel indirect proof of gene doping. Total RNA from mice (Con., N = 6; AAV-h*EPO*, N = 6) in the short-term experiments was extracted from 100 µL of whole blood using RNAiso Blood (Cat# 9112; Takara Bio) according to the manufacturer’s instructions. The RNA pellets were dissolved in 30 µL Milli-Q water, and the RNA solutions of 12 samples (Con.: n = 6; AAV-h*EPO*: n = 6) were tested for integrity using the Agilent RNA 600 Nano Kit (Cat# 5067-1511; Agilent Technologies, Santa Clara, CA, USA) on a bioanalyzer (Agilent Technologies). The RNA Integrity Number (RIN) of all samples was 9.5 or higher; thus, the RNAs of all eight samples could be subjected to library preparation for total RNA-seq. Libraries were prepared using 500 ng of RNA from each sample using the NEBNext Ultra II RNA Library Prep Kit for Illumina and the NEBNext rRNA Depletion Kit v2 (Cat# E7770S and E7400L; New England Biolabs, Ipswich, MA, USA) according to the manufacturer’s instructions. The final PCR cycle was 15. Concentrations and size distributions of the libraries were measured using an Agilent DNA 7500 Kit (Cat#5067-1506; Agilent Technologies, Santa Clara, CA, USA) with a Bioanalyzer. All samples were sent for analysis on NGS instruments. Libraries were pooled, and concentrations were adjusted to 1 nM. The pooled libraries were denatured and neutralized. The libraries were then diluted to 1.8 pM and subjected to an NGS run using NextSeq500/550 v2.5 (75 cycles) kits (Cat#20024906; Illumina, San Diego, CA, USA) on the NextSeq 500 System (Illumina). Sequencing was performed using 36-base paired-end reads. After the sequencing run, FASTQ files were exported, and the basic information of the NGS run data was checked using the CLC Genomics Workbench 24.0 software package (QIAGEN, Hilden, Germany). The quality of the reads was assessed. A total of 99.65% of the reads had a PHRED score greater than 20, indicating a successful run. The number of reads was approximately 11.4 to 16.1 million per sample as paired-end reads [8].

### 2.11. Bioinformatics Analysis

The following analysis was conducted to identify RNA markers (genes) as novel indirect proofs based on the concept of the ABP using NGS run data. FASTQ files were mapped to the mouse genome (GRCm39) using the CLC software (QIAGEN). A statistical differential expression test was performed using the Differential Expression in Two Groups tool in the software package. A principal component analysis (PCA) plot was created using the CLC software. Transcripts per kilobase million (TPM) or counts per million mapped reads (CPM) values were employed as an expression value for figure visualizations. A cluster dendrogram was generated using the R programming language (version 4.1.1). A tool for enrichment analysis was performed using Gene Set Enrichment Analysis (GSEA; version 4.3.3) with Mouse MSigDB (a database for enrichment analysis; mouse-ortholog hallmark gene sets; v2022.1.Mm) [23,24]. The Venny ver.2.1.0 [25] web tool was used to identify RNAs with dramatically fluctuating expression. Supplementary Table S2 presents the quantitative expression values, fold changes, and *p*-values of all genes obtained by analysis using the CLC software.

### 2.12. Measurements of mtDNA Copy Numbers in the Whole Blood

The results from the GSEA analysis of the above bioinformatics analysis showed that the gene set related to mitochondrial activity was significantly hit, so we checked the consistency of the results. The number of mitochondrial DNAs (mtDNAs) was measured using the whole-blood DNAs that were obtained from mice in the short-term experiment. The TB Green qPCR Assay was employed as described in Section 2.5. Primer pairs were designed to target nuclear DNA (nDNA) and mtDNA (Supplementary Table S1). The number of mtDNAs per nDNA was quantified using CT values obtained from the qPCR assay.

### 2.13. TB Green qPCR Assay for Whole-Blood RNA

To ascertain the reproducibility of the identified genes as indirect proof of gene doping, a TB Green qPCR assay was conducted on all samples (control: n = 8; AAV9-h*EPO*: n = 8) in the short-term experiment. RNA extraction from 100 µL whole-blood samples was performed using the same methods described in the “Total RNA-seq” Section. Subsequently, a TB Green qPCR assay was performed to assess the identified genes’ status as indirect proof using an identical methodology described in the “TB Green qPCR assay for tissue RNAs” Section. Primer sequences utilized in this section are presented in Supplementary Table S1. Furthermore, the period during which the identified indirect proof could be positively detected was investigated by repeating the same qPCR assay with 50 µL whole-blood samples (n = 11) from the aforementioned long-term experiment. Thereafter, the expression values of each targeted gene were normalized by the B2m (Beta 2-microglobulin) gene expression value [8].

### 2.14. Statistical Analysis

All data, with the exception of the total RNA-seq data, were subjected to statistical analysis using GraphPad Prism version 10.2.0 (GraphPad, San Diego, CA, USA). Prior to this, the Shapiro–Wilk normality test was employed to ascertain the normality of the distributions. Subsequently, nonparametric tests were employed for all data. Comparisons between three or more groups were conducted using the Kruskal–Wallis H test (one-way ANOVA of ranks), which is analogous to the one-way ANOVA. This test was followed by the two-stage Benjamini, Krieger, and Yekutieli FDR procedure, which is a post hoc test. A *p*-value less than 0.05 was considered to indicate statistical significance. All graphs that do not include data from the bioinformatics analysis of the total RNA-seq are presented as individual plots and medians with interquartile ranges.

## 3. Results

### 3.1. The rAAV9-hEPO Vector Worked toward Establishing a Mouse Model of Gene Doping

**Figure 2:**
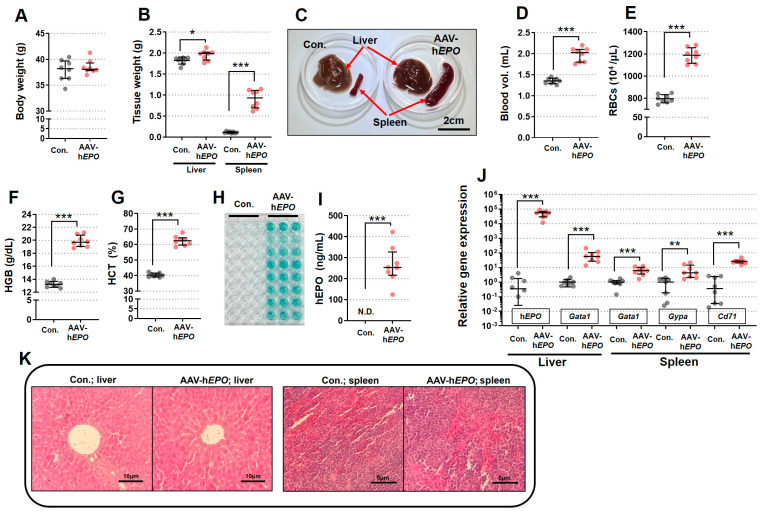
Establishment of a mouse model of gene doping with the rAAV9-h*EPO* vector. (**A**) Body weight; (**B**) tissue weight of liver and spleen; (**C**) external view of tissue of representative samples; (**D**) blood volume; (**E**) RBCs; (**F**) HGB; (**G**) HCT; (**H**) ELIZA reaction plates (N = 8 per group; measured in triplicate); (**I**) quantification of plasma hEPO by ELISA; (**J**) gene expression analysis of hematopoietic marker genes in each tissue; (**K**) pathological specimen images of liver and spleen (HE staining). N.D.: not detected. * *p* < 0.05, ** *p* < 0.01, *** *p* < 0.001.

Compared to the control group, the AAV-h*EPO* group showed a significant enlargement of the spleen and a slightly enlarged liver (Figure 2A–C), even though there was no change in body weight. In addition, blood tests showed a significant increase in blood volume, RBCs, HGB, and HCT in the AAV-h*EPO* group compared to the control group (Figure 2D–G).

ELISA demonstrated positive reactions (green coloration in the reaction plate; triplicate measurements; Figure 2H) in all mice of the AAV-h*EPO* group, with quantitative values ranging from 124 to 422 ng/mL (Figure 2I). Gene expression analysis by TB Green qPCR assay measured three hematopoietic marker genes (*Gata1*, *Gypa*, and *Cd71*) and h*EPO* genes in the liver and spleen tissue. The results showed that all-gene expressions were significantly increased in the AAV-h*EPO* group compared to the control group (Figure 2J). Pathology specimens showed an increase in nuclear size in both the liver and spleen in the AAV-h*EPO* group compared to the control group (Figure 2K). These results suggested that the rAAV9-h*EPO* vector worked well and was taken up by the mouse liver, where hEPO was secreted to the blood and hematopoiesis was actively occurring in the spleen. In other words, mice that mimic gene doping with the rAAV9-h*EPO* vector could be established.

### 3.2. The Four Primer/Probe Pairs Could Detect Direct Proof of Gene Doping

**Figure 3:**
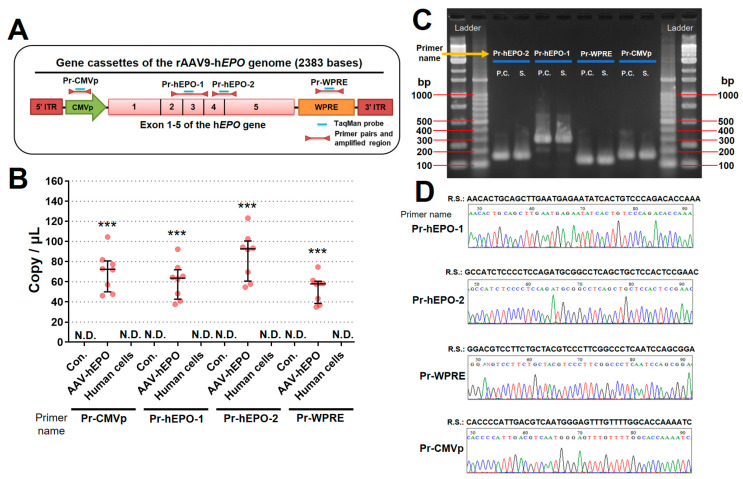
Detection of direct proof of gene doping in whole-blood DNA. (**A**) Designing strategy of primer/probe pairs for the rAAV9-h*EPO* vector; (**B**) detection of the direct proof using four primer/probe pairs; (**C**) gel electrogram of the amplicons; (**D**) waveforms and sequences of Sanger sequences using the amplicons as templates. N.D.: not detected; P.C.: positive control; S.: samples; R.S.: reference sequence. *** *p* < 0.001 vs. Con. or human cells.

The strategies to create four primers/probes are shown in Figure 3A. All primers/probes designed in this study worked and were highly sensitive, with a lower limit of 0.95 copies (viral genome)/μL. In addition, the viral DNAs were not detected in whole-blood DNAs from control mice or in human cell line DNAs. In contrast, the AAV-h*EPO* group showed positive PCR amplification with a copy number of 34-104 copies (viral genome)/μL (Figure 3B). The amplified products (AAV-h*EPO* mouse and positive control only) after the qPCR assay were confirmed by electrophoresis, and all lanes showed amplified products of the predicted size (Figure 3C). The amplified products (AAV9-h*EPO* mice only) after their qPCR were confirmed by Sanger sequencing, the waveform data were correctly depicted, and the obtained sequence matched the target sequence (Figure 3D). These results suggest that these primer/probe pairs and the qPCR assay worked and were able to accurately detect proof of gene doping.

### 3.3. The Total RNA-Seq Revealed Drastic Changes in RNA Expression and Identified Several Genes That Provide Indirect Proof of Gene Doping

**Figure 4:**
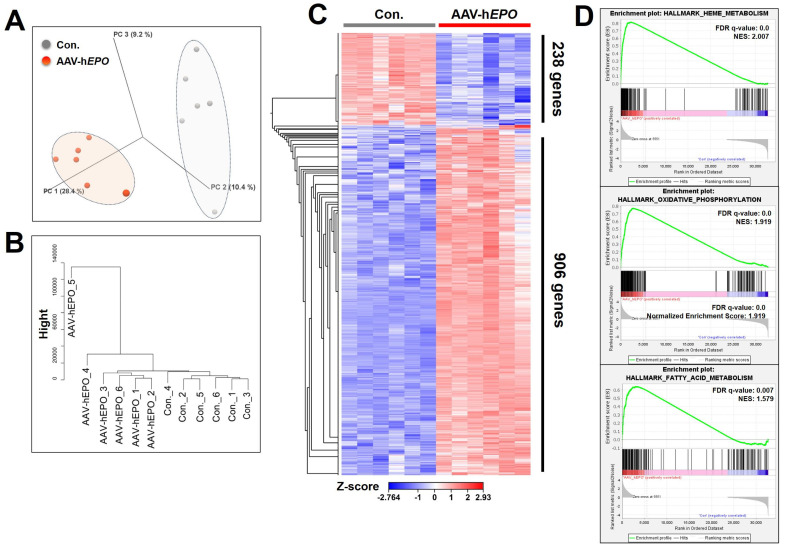
Understanding the results and overall gene expression of total RNA-seq and bioinformatics analysis. (**A**) PCA plot; (**B**) cluster dendrogram; (**C**) heatmap for the 1144 genes; (**D**) results of the top-three gene sets on the GSEA analysis. NES: normalized enriched score.

**Figure 5:**
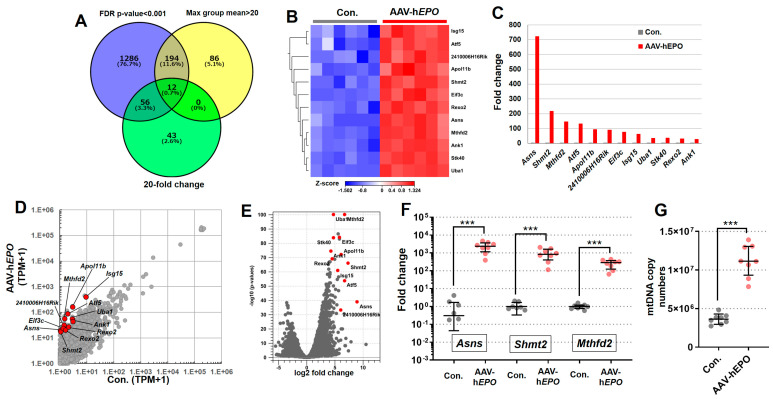
Identified 12 promising genes with drastic changes in expression. (**A**) Venn diagram analysis to identify the 12 genes; (**B**) heatmap of the 12 genes; (**C**) bar graph of the relative expression value (average of TPM value) of 12 genes; (**D**) scatter plot collared the 12 genes; (**E**) volcano plot collared the 12 genes; (**F**) TB Green qPCR assay for all mice (N = 8, respectively) of the top 3 genes. (**G**) mtDNA copy numbers. *** *p* < 0.001.

The results of the total RNA-seq using whole blood showed that the expression of many RNAs was drastically changed. In the 3D PCA plot and cluster dendrogram, clear distance and separation by clusters between Con. and AAV-h*EPO* groups suggested that the two groups were in different states of gene expression (Figure 4A,B). In the statistical comparison between the Con. and AAV-h*EPO* groups, a total of 1144 genes were found to have significant variation when filtering for an FDR *p*-value < 0.001, 2-fold change, and max group mean > 1 as thresholds. Of those 1144 genes, the expression of 906 genes was increased in the AAV-h*EPO* group, and that of the remaining 238 genes was decreased, as shown in the heatmap (Figure 4C). The results of the GSEA analysis showed that the gene sets of “heme metabolism”, “oxidative phosphorylation”, and “fatty acid metabolism” were significantly enriched due to upregulation in the AAV-h*EPO* group (Figure 4D). The analysis to this point has shown that gene-doped mice injected with the rAAV-h*EPO* vector showed substantial fluctuations in RNA expression in whole blood, reflecting characteristic enrichment in some gene sets. Further bioinformatics analysis was performed to identify single gene expression with dramatic variation. Using Venn diagram analysis, strict thresholds were set, namely an FDR *p*-value < 0.001, a 20-fold change, and a max group mean > 20, and then 12 promising genes were identified (Figure 5A). The expressions of these 12 genes were increased in the AAV-h*EPO* group compared to the Con. group, as shown in the heatmap (Figure 5B). Moreover, these genes were analyzed and visualized as a bar graph (average value of the TPM; Figure 5C), scatterplot (Figure 5D), and volcano plot (Figure 5E). Consistent results were then obtained. In particular, *Asns* (*asparagine synthetase*), *Shmt2* (*serine hydroxymethyltransferase 2*), and *Mthfd2* (*methylenetetrahydrofolate dehydrogenase 2*), a top-three gene, showed a more than 100-fold fluctuation (Figure 5C). TB Green qPCR assays were performed on these three genes to confirm reproducibility against the total RNA-seq in all mouse samples. The results were generally consistent (Figure 5F). As per the result of the GSEA analysis, mitochondrial activation of the whole blood was suspected in the AAV-h*EPO* group. Moreover, *Shmt2* and *Mthfd2* are known to be associated with mitochondrial function. To confirm their consistency, mtDNA copy numbers were quantified, and then it was found that the copy numbers were significantly increased in the AAV-h*EPO* group compared to the Con. group. Taken together, these results suggest that inducing the h*EPO* transgene in the rAAV9 vector alters the biological significance of whole-blood RNA and causes some genes to be dramatically upregulated with increasing mitochondrial numbers, which has the possibility of becoming an ABP parameter.

### 3.4. Proof of Gene Doping Was Detectable in about a Drop of Whole Blood for 30 Days

**Figure 6:**
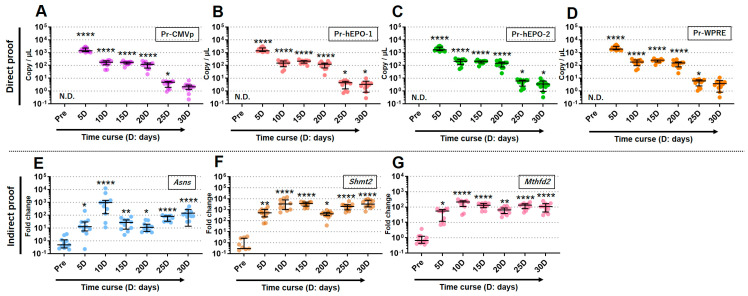
Detection of direct or indirect proof of gene doping for 30 days. (**A**–**D**) Detection of direct proof using each primer–probe; (**E**–**G**) detection of indirect proof as three gene expressions leading to the concept of ABP. * *p* < 0.05, ** *p* < 0.01, **** *p* < 0.0001 vs. Pre, respectively.

The qPCR assays were performed to detect direct or indirect proof of gene doping using micro blood samples (about 50 uL; one drop) from the tail taken repeatedly over a period of 30 days. The statistically significant detection of direct proof of gene doping with each of the primers/probes was possible up to day 25 for Pr-CMVp and Pr-WPRE and up to day 30 for Pr-hEPO-1 and Pr-hEPO-2 (Figure 6A–D). Moreover, the copy numbers of the rAAV-h*EPO* genome as direct proof peaked at day 5 for all primers/probes and then unidirectionally decreased until day 30 (Figure 6A–D).

The expression values of three genes that could be indirect proof of gene doping, thus leading to the ABP concept, were significantly elevated in all periods until day 30 (Figure 6E–G). On the other hand, unlike the dynamics of direct proof, the expression level remained generally constant between day 5 and day 30. These results indicated that direct or indirect proof of gene doping could be detected using a single drop of whole blood.

## 4. Discussion

The first objective of this study was to create a mouse model that mimics gene doping with confirmed hematopoietic effects using rAAV9 carrying the h*EPO* gene. rAAV9, when injected into a mouse vein, accumulates in the liver and induces long-term expression of the target gene [26]. It has also been confirmed that the period of continuous expression is more than 9 months [26]. Therefore, in this study, it can be assumed that the injected rAAV9 reached the mice's liver and induced h*EPO* gene expression in the hepatocytes over a long-term period. In support of this speculation, gene expression of h*EPO* was detected in the liver. The h*EPO* gene contains a secreted signal peptide, so the translated protein is also expected to circulate throughout the whole body as a hormone via the blood. In fact, an ELISA confirmed the presence of human erythropoietin in the AAV-h*EPO* group, confirming that it circulates throughout the body. In addition, the main hematopoietic organ in adult mice is the spleen [27]. In this animal experiment, the spleen of mice was enlarged, and the expression of hematopoietic marker genes was significantly increased. Moreover, RBCs, HGB, HCT, and blood volume, which directly reflect hematopoietic effects, were also significantly increased in the AAV-h*EPO* group. Taken together, these results suggest that the rAAV9-h*EPO* vector induces the expression of h*EPO* in the liver after intravenous injection and that the hEPO hormone reaches the spleen through the bloodstream, thereby upregulating hematopoiesis. Therefore, the first objective in this study has been achieved, meaning that the establishment of a biological model of gene doping using AAV and h*EPO* genes has been accomplished for the first time. In previous studies on gene doping tests targeting h*EPO*, detection methods were developed by artificially mixing plasmids with blood as a spike-in test [28,29,30,31]. On the other hand, our model, however, uses an actual living organism and can consider the metabolic state of the vector, so it has the potential to be developed into a more practical model. Therefore, in the future, we plan to provide various technical support so that many researchers around the world can use our models with good reproducibility.

In this study, four specific primers/probes were generated to target DNA sequences in rAAV9-h*EPO* as direct proof of gene doping. For the h*EPO* gene, we designed the probe to span the exon–exon junction in accordance with WADA guidelines [21]. Although the WADA guidelines do not mention the use of multiple primers/probes, the use of this method may increase the scientific validity and accuracy of the test. In addition, the use of multiple primers/probes would allow for an estimation of the type of vector used. However, in this study, one qPCR assay was performed on one target. In the future, it would be desirable to establish a multiplex assay and develop a kit with a panel comprising each vector and gene. This would reduce the testing time and cost. In addition, the four primer probes used in this study detected positive reactions only in mice induced with the rAAV9-h*EPO* vector, with no nonspecific amplification from human DNAs or an NTC (non-template control). An analysis of the standard curve showed that the detection limit was approximately 1 copy/uL, indicating the high detection sensitivity of these primers/probes. Therefore, in the future, it may be possible for these four primers/probes to be directly used for gene doping testing using blood DNA samples from athletes.

Based on the ABP concept proposed by WADA [9], we hypothesized and tested the hypothesis that RNA (gene expression) in whole blood may be targeted as a new ABP marker. As a result, in the total RNA-seq, we found that mice induced with the rAAV9-h*EPO* vector showed significant changes in the expression of many RNAs in whole blood, and heme metabolism, oxidative phosphorylation, and fatty acid metabolism were significantly enriched in terms of their biological significance and pathways in terms of their gene sets. Further bioinformatics analysis identified three dramatically upregulated RNAs (genes), which remained highly expressed after 30 days. These results suggest that pathway analysis using a specific set of genes or analysis for single RNA markers can be very useful as a measure of ABP. However, the total RNA-seq performed in this study is expensive in terms of reagents and analysis costs, and the equipment required is also expensive. Although the application of RNA-seq technology to ABP testing is promising, the cost and difficulty of the technology may be prohibitive. Therefore, cost reductions and technological innovations are eagerly awaited.

If this were translated to humans, it would be possible to detect genetic doping with only a trace amount of blood (one drop of blood) drawn from a fingertip after a competition. If the amount of specimen is only one drop, the development of small testing devices may also enable rapid testing for genetic doping in the field. The closest social implementation would be a PCR test for the Severe Acute Respiratory Syndrome Coronavirus 2 (SARS-CoV-2) infection, also known as the COVID-19 pandemic, which has raged in recent years. With the current proliferation of various simplified test reagents and small qPCR devices, rapid PCR testing has been implemented in the field around the world. These technologies are probably very compatible with on-site rapid tests for genetic doping. Therefore, a future may not be far off in which primary on-site screening for genetic doping will be available for many athletes.

It is important to mention that there are several limitations to this study. Although this study was the first in the world to establish a mouse model of gene doping using the rAAV9 vector, including the h*EPO* gene, and the first to develop a detection method, it is only a mouse model and cannot yet be said to be applicable to actual human athletes. In future studies, the most accurate findings could be obtained by using blood samples from actual gene-doped athletes, but this is not possible due to legal or ethical aspects. As an alternative, it would be most reasonable to validate the results of this study using surplus blood samples from patients undergoing gene therapy with rAAV. As indicated in the database, many clinical trials using rAAV are being conducted around the world [19]. In addition, several Food and Drug Administration (FDA)-approved rAAV therapies [32,33,34,35] have become widely available in recent times. Research and development to prevent gene doping would require collaboration with patients receiving rAAV medications and their healthcare providers, as well as clinical trials. In doing so, the findings of this study would be helpful in creating safe experimental protocols and setting conditions for detection methods.

This study has one limitation. Only one dose of the rAAV9-h*EPO* vector was administered in this mouse experiment. This dose has been previously validated in other studies [26] and would be considered standard practice for researchers working with rAAV vectors in laboratories. Furthermore, medical doses of rAAV for humans have been reported to be between 2 × 10^11^ and 2 × 10^14^ vector genomes per kilogram (vgs/kg) per patient [18], which is considered to remain within a reasonable range when considered in terms of mouse body weight. In our pilot experiment using fewer mice, the hematopoietic effect was not sustained from day 20 to day 25 at doses of 1/4 to 1/8 of the dose determined in this study (unpublished data). Consequently, the dose determined in this study may be a viable and reasonable dose. However, the suitability of this dosage cannot be definitively determined from the present study. Further, detailed experiments must be conducted to resolve this issue. Ideally, each parameter should be analyzed in a dose-dependent manner, and the detection of gene doping should be verified. The next step is to address these issues and develop a more robust mouse model and test methods.

## 5. Conclusions

In this study, we were able to establish gene-doping model mice using the rAAV9-h*EPO* vector. Furthermore, not only were we able to detect direct proof of gene doping in whole blood in these mice, but we also revealed that RNA expression in whole blood fluctuates significantly, and we discovered that whole-blood RNA (gene) expression could become a new ABP parameter. The findings of this study will be beneficial for future research and development in this field.

## Data Availability

Data are contained within the article and Supplementary Materials.

## Supplementary Materials

The following supporting information can be downloaded at https://www.mdpi.com/article/10.3390/genes15060709/s1, Table S1: primers/probes; Table S2: Expression browser.
